# Cognitive Function and Mortality: Results from Kaunas HAPIEE Study 2006–2017

**DOI:** 10.3390/ijerph17072397

**Published:** 2020-04-01

**Authors:** Abdonas Tamosiunas, Laura Sapranaviciute-Zabazlajeva, Dalia Luksiene, Dalia Virviciute, Martin Bobak

**Affiliations:** 1 Institute of Cardiology, Medical Academy, Lithuanian University of Health Sciences, LT-50162 Kaunas, Lithuania; abdonas.tamosiunas@lsmuni.lt (A.T.); dalia.luksiene@lsmuni.lt (D.L.); dalia.virviciute@lsmuni.lt (D.V.); 2 Health Psychology Department, Lithuanian University of Health Sciences, LT-47181 Kaunas, Lithuania; 3 Institute of Epidemiology and Health Care, University College London, London WC1E 7HB, UK; m.bobak@ucl.ac.uk

**Keywords:** cognitive functions, cardiovascular mortality, all-cause mortality

## Abstract

Background: The purpose of the study is to evaluate the association between cognitive function and risk of all-cause and cardiovascular disease mortality during 10 years of the follow-up. Methods: 7087 participants were assessed in the baseline survey of the Health Alcohol Psychosocial Factors in Eastern Europe (HAPIEE) study in 2006–2008. During 10 years of follow-up, all-cause and CVD mortality risk were evaluated. Results: During 10 years of follow-up, 768 (23%) men and 403 (11%) women died (239 and 107 from CVD). After adjustment for sociodemographic, biological, lifestyle factors, and illnesses, a decrease per 1 standard deviation in different cognitive function scores increased risk for all-cause mortality (by 13%–24% in men, and 17%–33% in women) and CVD mortality (by 19%–32% in men, and 69%–91% in women). Kaplan-Meier survival curves for all-cause and CVD mortality, according to tertiles of cognitive function, revealed that the lowest cognitive function (1st tertile) predicts shorter survival compared to second and third tertiles (p < 0.001). Conclusions: The findings of this follow-up study suggest that older participants with lower cognitive functions have an increased risk for all-cause and CVD mortality compared to older participants with a higher level of cognitive function.

## 1. Introduction

Older people often experience cognitive deficits such as memory loss and attention disturbances that can interfere with daily functioning [1,2,3,4,5]. Previous studies found that poor cognitive function predicts mortality in an older population [6,7]. Researchers revealed that even low levels of cognitive function impairment not reaching the level of dementia increases the risk of mortality [5]. It is suggested that subjective cognitive impairment, which is recognised as a potential indicator for cognitive decline, is linked to all-cause mortality [8]. However, some studies argue that subjective cognitive decline does not predict mortality when objective cognitive deficits are controlled [9,10,11]. As scientists put it, complaints about cognitive abilities do not ultimately lead to cognitive impairment in all cases [10,11]. Some authors stated that cognitive impairment does not affect mortality without physical disability [12].

It was proposed that cognitive function decline is related to mortality, even independently of the initial level of cognitive abilities [13]. The mechanisms under this link are still unknown, but might include a decreased ability to follow the medical regime and self-care. It also might be the result of the lack of social integration [14,15]. Cognitive deficits in older age are often associated with vascular cognitive impairments. Cognitive deficits from mild cognitive impairment to dementia share the same risk factors such as low educational level, smoking, diabetes, and depression with other cardiovascular diseases [16,17]. However, not only is the negative influence of the cognitive deficit being analyzed, but good cognitive trajectories have also been associated with decreased mortality [18].

However, the link between cognitive function and mortality is not clear. Previous studies ascertained different links between cognitive decline and survival [9]. There also might be a difference in association according to the cause of death [19]. Some previous studies established that cognitive function is linked with all-cause mortality but not cancer death [1,18]. Another study found that cognitive decline, on the contrary, decreases the risk of cancer mortality [20]. Moreover, previous studies found that specific cognitive functions such as short-memory or cognitive speed may be differently associated with mortality than the total cognitive score [21,22]. This study explored associations between different cognitive functions and different causes of death accounting for significant covariates in the representative sample.

## 2. Materials and Methods

### 2.1. Study Population and Design

This data was part of the Health, Alcohol, and Psychosocial Factors in Eastern Europe (HAPIEE) study baseline survey and follow-up for endpoints in Kaunas (Lithuania) [23]. A total of 10,940 men and women who were 45–72 years of age were randomly selected from the National Population Register of Lithuania. Of them, 7087 (64.8%) individuals participated in this survey from 2006 to 2008. All participants were followed up for all-cause and cardiovascular disease (CVD) mortality events until 31 December 2017. The study design, the methodology of measurements, variables determined using the questionnaire, and definitions of the risk factors and health conditions were described in detail in our previous publication [24]. We excluded 183 respondents from the statistical analysis sample because of incomplete information on study variables. Furthermore, 1509 (21.3%) participants with CVD at baseline (ischemic heart disease (IHD) or stroke) were not included in the analysis of CVD mortality risk. The final number of participants included in the analysis was 6904 evaluating for all-cause mortality, and 5395 for CVD mortality. The study was approved by the Ethics Committee at University College London, UK, and by Kaunas Regional Biomedical Research Ethics Committee.

### 2.2. Cognitive Function

The cognition battery consisted of five tests to assess performance across different cognitive domains: immediate and delayed verbal memory, semantic verbal fluency, speed and concentration, and numerical ability. We also calculated the composite score of cognitive function. Scores representing a composite score of cognitive function were assessed by averaging z-scores for each test and summing the results. Lowered cognitive function was determined using a composite score of cognitive function. Low cognitive status shows high levels of impairment and greater decline in cognition. However, high cognitive status means low impairment. The participants with a composite score value 1 standard deviation (SD) or more below their age and education-specific means of cognitive function were classified into a lowered cognitive function group. The assessment of cognitive function, the procedures of cognitive function tests, calculation of the composite score of cognitive function, and definition of lowered cognitive function were previously described in detail [24].

### 2.3. Covariates

We included statistical analysis as covariate variables determined using the standard questionnaire (age, education, marital status, depressive symptoms, and lifestyle factors (smoking habits, physical activity, and alcohol consumption)), measurements (blood pressure, body weight, and height), and laboratory analyses (levels of total cholesterol, high-density lipoprotein (HDL) cholesterol, low-density lipoprotein (LDL) cholesterol, triglyceride, and fasting glucose). The variables and their classification are described in detail in our previous article [24]. Diabetes and CVD (IHD and stroke) at baseline were also used as covariates in statistical analyses of the study data. IHD or stroke were not included in the analysis of CVD mortality risk. The definitions of the mentioned health conditions were also described previously [24].

Additionally, for the indicated covariates, we used the variable of psychological well-being (PWB). PWB was determined by a Control Autonomy Self-Realisation and Pleasure (CASP-12) questionnaire composed of 12 statements [25]. The total PWB score could vary from 12 to 48. The participants were classified as having lower PWB if the CASP-12 score was lower than the median: <40 in men and <38 in women. The low PWB score shows a subjective perception of a low capacity for self-care and self-realization. A more precise presentation of PWB has been described previously [26].

### 2.4. Mortality

Data from the Kaunas Mortality Register based on death certificates were used for the registration of the death events in the study participants during the follow-up period. Causes of death were coded by the International Classification of Diseases (ICD) (version 10): all causes of death included codes A00-Z99, and deaths of CVD included codes I00–I99.

### 2.5. Statistical Analysis

Descriptive characteristics (prevalence rates, means, and standard errors (SE)) were calculated for variables in groups of a vital status at the end of the follow-up period (alive and dead from all-causes and CVD) separately for men and women. The differences in age-adjusted means of variables between the alive and dead groups were assessed using T-test and ANOVA analysis with Bonferroni multiple comparison tests. A chi-squared test and z test with Bonferroni corrections were used for assessing the differences in categorical variables. *p* < 0.05 values were considered statistically significant. The Kaplan-Meier survival curves for cumulative all-cause mortality and CVD mortality according to the tertiles of a composite score of cognitive function for men and women were plotted. A log-rank test was performed to compare the difference between cognitive function tertiles. Hazard ratios (HR) and 95% confidence intervals (CI) were estimated by the Cox proportional hazards’ regression for CVD and all-cause mortality. The risk of all-cause and CVD mortality was calculated per each 1 standard deviation (SD) decrease of a composite score of cognitive function, and other cognitive function tests (immediate verbal recall sum, delayed verbal recall, semantic verbal fluency, numerical ability, and cognitive speed and attention). Four models were assessed. Model 1 was adjusted for age (continuous variable), education, and marital status (categorical variables). Model 2 adjusted for all the variables in Model 1 plus lifestyle (smoking—categorical, physical activity in leisure (continuous—hours/week), alcohol consumption (continuous—drinks/week), and biologic factors (systolic or diastolic blood pressure—continuous, total cholesterol, high density lipoprotein (HDL) cholesterol, low density lipoprotein (LDL) cholesterol, triglycerides, fasting glucose, body mass index (BMI)—all continuous). Model 3 was adjusted for all the variables in Model 2 plus depression symptoms (categorical) and PWB (continuous). Model 4 adjusted for all the variables in Model 3 plus existing illness (IHD, diabetes, and stroke—for all-cause mortality; diabetes—for CVD mortality (all categorical)). Statistical analysis was performed using SPSS version 20.0 (IBM Corp., Armonk, NY, USA).

## 3. Results

The study participants were followed-up from the beginning of the baseline health examination date until December 31st, 2017. The mean duration of follow-up was 9.5 ± 2.52 years (9.1 ± 2.83 years among men, and 9.83 ± 2.19 years among women). Over the follow-up period, 1051 (14.3%) death cases from any cause (667 men, and 384 women) and 460 (6.2%) deaths from CVD (307 men, and 155 women (160 men and 81 women when previous CVD events at entry were excluded)) were registered.

The baseline characteristics of respondents according to vital status are shown in Table 1. Men and women who died from all-cause deaths and CVD deaths during the follow-up period were significantly older, had lower level of education, had a higher proportion of widowers and a lower proportion of cases who were married than those alive at the end of the follow-up. The aged-adjusted means of some biologic factors, such as systolic and diastolic blood pressure and fasting glucose level were higher in men and women who died during the follow-up period compared to those who were alive. However, some biological factors differed due to gender and cause of mortality. Moreover, the men and women who died had higher IHD, stroke and diabetes mellitus rate than those alive at the end of the follow-up. The prevalence of lifestyle habits also differs according to gender and vitality. Women who died had a higher proportion of physical inactivity and a higher level of BMI. Among men, the number of smokers was higher in those who died compared to those who were alive. The men and women who died (all-cause deaths and CVD deaths) during the follow-up period had a lower cognitive function, PWB, and more depressive symptoms than the living participants at the end of the follow-up. Additionally, their age-adjusted means of cognitive functions were lower than among the alive individuals. Variables of cognitive function, among participants who died, such as immediate verbal recall, delayed verbal recall, semantic verbal fluency, numerical ability, cognitive speed and attention, and a composite score of cognitive function were significantly lower than among the alive individuals at the end of the follow-up.

Table 2 presents the risk of all-cause and CVD mortality in men and women after adjustment for socio-demographic, lifestyle, and biological risk factors, existing illness, depressive symptoms, and PWB. We evaluated the risk for all-cause mortality and CVD mortality concerning the scores of various cognitive functions. After adjustment for socio-demographic factors (Model 1), a decrease per 1 SD in the scores of cognitive functions, and the composite score of cognitive function significantly increased the risk for all-cause mortality (by 17–33% in men, and by 20–43% in women) and CVD mortality in men (by 24–48%) and women (by 35–81%). However, a decrease in numerical ability increased the risk only for CVD mortality and only in the male group. After additional adjustment for lifestyle and biological risk factors, depressive symptoms, and PWB (Model 2 and Model 3), the risk for all-cause mortality remained statistically significant in men and women, as well as the risk of CVD mortality in women. In men, such a significant relationship was not determined for immediate verbal recall and semantic verbal fluency. The relationship between the cognitive function and the risk for all-cause mortality and CVD mortality remained statistically significant for most tests after adjusting for one more additional factor—existing illnesses (Model 4).

**Figure 1:**
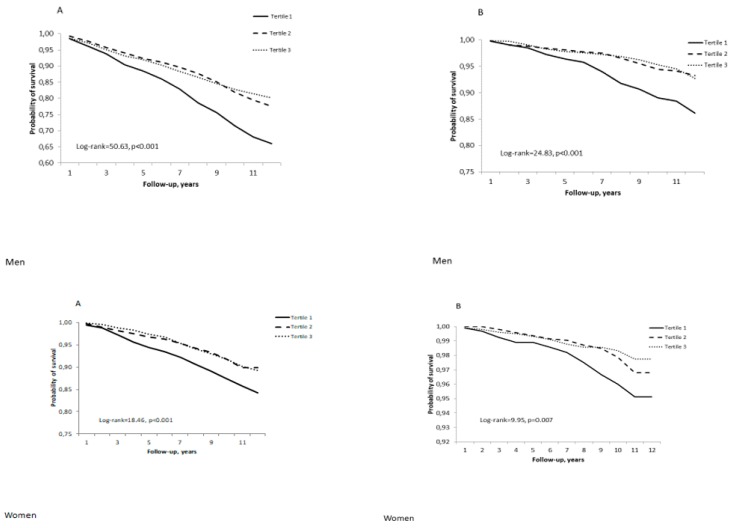
Kaplan-Meier survival (all-cause (**A**) and cardiovascular (**B**) mortality) curves according to the tertiles of a composite score of cognitive function for men and women.

Kaplan-Meier survival curves for cumulative all-cause mortality (A) and CVD mortality (B) according to the tertiles of a composite score of cognitive function for men and women, adjusted for age, are presented in Figure 1.

The log-ranked test revealed that the cumulative survival rates within 10 years of follow-up significantly differ (*p* < 0.01) for three levels of the composite score of cognitive function. The lowest tertile of a composite cognitive function score predicts shorter survival compared with the second and the third tertile of the composite score of cognitive function (among men, the survival rates were 71.4%, 81.7%, and 82.8% while, among women, the survival rates were 86.9%, 91.3%, and 91.4%, respectively).

## 4. Discussion

The current study analyzed the links between different cognitive functions and the risk of death. Poor cognitive functions were associated with all-cause and CVD mortality during 10 years of follow-up. Data showed that the risk of mortality was higher among persons with high cognitive impairment compared to low cognitive impairment, even after controlling for confounding, as in previous studies [27]. Links were ascertained not only for a composite score of cognitive functions, but also for separate cognitive abilities, as in previous research studies [28].

A decrease in scores of immediate and delayed verbal recall, semantic verbal fluency, cognitive speed, and attention was associated with increased risk of mortality in men and women. Only a numerical ability score was not associated with mortality risk. The decrease in the score of cognitive speed was the best predictor of mortality in men and women, compared with the decrease in other domains and even with a decrease of the composite cognitive function score. A decrease in immediate and delayed verbal recall, semantic verbal fluency, cognitive speed, and attention increased the risk of mortality in men and women. Numerical ability was not associated with mortality risk. Previous studies ascertained that poor cognitive speed was associated with mortality risk even in younger males [21]. As previous studies stated, not all the cognitive functions have the same effect on mortality [3].

Cognitive functions are associated with increased mortality risk. However, the mechanisms remain obscure [20]. Cognitive abilities are associated with health literacy, as people with lower cognitive function may be less able to engage in a healthy lifestyle and are less able to follow specialist instructions [19,29]. Additionally, people with lower cognition are unable to engage in a healthy lifestyle [22,30]. Moreover, cognitive functions might affect health as a part of a multi-dimensional concept like frailty, where it works along with other factors such as exhaustion, weight loss, etc. [31,32,33]. Higher impairment of cognitive function may reduce the survival rate because of the beginning processes of the pathophysiology of dementia [32,34,35]. It is very important to mention the possible pathway through vascular cognitive impairment, where the role of micro-infarcts, micro-hemorrhages, strategic white matter tracts, loss of microstructural tissue integrity, and secondary neurodegeneration becomes crucial [17]. Moreover, the prevention of cardiovascular diseases such as coronary artery disease and chronic heart failure lowers the risk of vascular cognitive impairment [36]. However, it is presently unclear how low levels of cognitive impairment might proceed from mild cognitive impairment to dementia and mortality. In some people, mild cognitive impairment is connected to dementia. However, for others, it is more commonly connected to a general decline in health [37].

The study established that mortality for decreased cognitive functions differs by gender. The effect was higher for women than men. A previous study established that a 5-year mortality risk increased among women with mild cognitive impairment and dementia compared to women with normal cognition, which accounts for traditional prognostic indicators [7]. Some studies did not find a significant difference between men and women in this association [13]. Another previous study ascertained that the risk was higher in the male group [15].

There are also differences ascertained due to cause of death. The risk of all-cause mortality is less significant compared to CVD mortality, according to the decrease of cognitive functions. The previous study also found that the strength of this relationship differs due to the cause of death [19]. A strong link between CVD death and cognitive functions might be due to common cardiovascular risk factors [2,15].

Survival curves revealed that mortality risk increases in the lowest tertile of the composite the cognitive function score compared with the first and second tertile. Previous studies revealed that the mortality risk increases in parallel with the severity of impairment. Therefore, the risk is high in cases of mild cognitive impairment and not significant in cases of questionable impairment [38]. Thus, mortality risk was significant only for people in the lowest tertile of cognitive function.

Some strengths should be mentioned in the context of this study. This is the first study in the Baltic region that analyzes the link between cognitive functions and mortality in older adults. Our study contains a large, representative sample. It analyzed the association between mortality and cognitive functions in different models. This reduces bias by controlling socio-demographic, psychological, and biological CVD risk factors. Previous studies noted that, in the analysis of cognitive function and mortality association, various covariates should be controlled, such as physical and mental health factors as well as psychosocial [37] and cerebrovascular risk factors [39,40,41]. We assessed the various components of cognitive function, such as immediate and delayed memory, attention and cognitive speed, and not only the composite score as usual. The contribution of this study to current literature on the subject is that numerical ability did not predict mortality and that the cognitive speed predicted mortality even better than the composite score. It might be considered in future studies to administer the cognitive speed test, rather than the entire battery of cognitive tests. Moreover, links were ascertained with all-cause mortality and a specific cause of mortality. Our study revealed that cognitive deficit better predicted CVD mortality than all-cause mortality.

Many factors that might affect the link between cognitive function and survival were controlled. However, possible confounding might remain and attenuate the link. Other factors such as genes [39,42], physical disability [12], and frailty [31,32,33], which can lead to cognitive decline and mortality but that were not measured in the survey, can contribute to unmeasured confounding factors. The limitation of our study is that the decline of cognitive function was not controlled during the follow-up since cognitive functions were tested only in the beginning of the study. Several biases may arise due to possible changes in cognitive function and other variables over the follow-up period. On the other hand, the purpose this study was not to evaluate the effect of cognitive decline to mortality, but rather to evaluate the effect of the initial cognitive function level on mortality during the 10 years of follow-up. Future studies should capture cognitive functions as a time varying covariate. The follow-up of the cognitive functions might be useful to capture the decline of the cognitive function and its effect on mortality.

## 5. Conclusions

Mortality risk was increased among older men and women with a higher level of cognitive impairment, which accounts for various prognostic indicators. However, some differences of associations were ascertained based on specific cognitive function, cause of death, and gender. Nonetheless, our findings suggest that cognitive function should be monitored over time to prevent cognitive decline and extend lifespan in older age.

## Figures and Tables

**Table 1:** Baseline characteristics of the Kaunas Health Alcohol Psychosocial Factors in Eastern Europe (HAPIEE) study sample according to vital status and sex at the end of the follow-up period (31 December 2017).

		MEN			WOMEN	
Variables	Alive	All-Cause Deaths	CVD Deaths ^c^	Alive	All-Cause Deaths	CVD Deaths ^c^
	n = 2462	n = 667	n = 160	n = 3391	n = 384	n = 81
Age, years	56.1 ± 0.15	62.0 ± 0.34 ^b^	62.7 ± 0.50 ^b^	56.3 ± 0.13	63.0 ± 0.46 ^b^	66.4 ± 0.53 ^b^
Immediate verbal recall sum ^a^, score	21.9 ± 0.08	19.5 ± 0.18 ^b^	19.1 ± 0.27 ^b^	23.3 ± 0.06	21.2 ± 0.25 ^b^	19.9 ± 0.41
Delayed verbal recall ^a^, score	7.72 ± 0.36	6.76 ± 0.09 ^b^	6.61 ± 0.14 ^b^	8.32 ± 0.27	7.39 ± 0.11 ^b^	6.93 ± 0.20 ^b^
Semantic verbal fluency ^a^	22.6 ± 0.12	20.0 ± 0.26 ^b^	19.4 ± 0.37 ^b^	22.4 ± 0.10	19.5 ± 0.38 ^b^	17.3 ± 0.55 ^b^
Numerical ability ^a^, score	3.14 ± 0.01	2.95 ± 0.04 ^b^	2.89 ± 0.06 ^b^	2.91 ± 0.01	2.71 ± 0.05 ^b^	2.57 ± 0.08 ^b^
Cognitive speed and attention ^a^	16.5 ± 0.09	13.7 ± 0.20 ^b^	13.2 ± 0.32 ^b^	17.6 ± 0.08	14.6 ± 0.30 ^b^	13.5 ± 0.52 ^b^
Composite score of cognitive function ^a^	–0.08 ± 0.01	–0.27 ± 0.03 ^b^	–0.34 ± 0.05 ^b^	0.11 ± 0.01	-0.06 ± 0.04 ^b^	–0.17 ± 0.07 ^b^
Systolic blood pressure ^a^, mm Hg	143.3 ± 0.40	150.9 ± 1.04 ^b^	153.7 ± 1.63 ^b^	133.4 ± 0.34	143.0 ± 1.43 ^b^	150.8 ± 2.47 ^b^
Diastolic blood pressure ^a^, mm Hg	92.4 ± 0.25	94.9 ± 0.61 ^b^	95.9 ± 0.93 ^b^	87.4 ± 0.20	90.8 ± 0.76 ^b^	93.0 ± 1.33 ^b^
Total cholesterol ^a^, mmol/L	5.83 ± 0.02	5.74 ± 0.05	5.80 ± 0.08	6.07 ± 0.02	5.96 ± 0.07	5.94 ± 0.12
HDL cholesterol ^a^, mmol/L	1.41 ± 0.01	1.40 ± 0.02	1.31 ± 0.02 ^b^	1.60 ± 0.01	1.51 ± 0.02 ^b^	1.46 ± 0.03 ^b^
LDL cholesterol ^a^, mmol/L	3.74 ± 0.02	3.64 ± 0.05 ^b^	3.73 ± 0.07	3.83 ± 0.02	3.73 ± 0.06	3.72 ± 0.10
Triglyceride^a^, mmol/L	1.51 ± 0.02	1.57 ± 0.05	1.76 ± 0.08 ^b^	1.40 ± 0.02	1.53 ± 0.05 ^b^	1.67 ± 0.10 ^b^
Fasting blood glucose ^a^, mmol/L	5.72 ± 0.02	6.05 ± 0.07 ^b^	6.29 ± 0.13 ^b^	5.75 ± 0.02	6.19 ± 0.10 ^b^	6.55 ± 0.21 ^b^
Body mass index ^a^, kg/m^2^	28.3 ± 0.09	28.5 ± 0.23	29.6 ± 0.36 ^b^	29.3 ± 0.10	31.5 ± 0.37 ^b^	31.9 ± 0.67 ^b^
Physical activity in leisure time ^a^, hours/week	17.1 ± 0.24	16.1 ± 0.59	15.5 ± 0.84	19.7 ± 0.19	17.1 ± 0.62 ^b^	16.7 ± 1.06 ^b^
Absolute alcohol ^a^, drinks/week	58.7 ± 2.13	65.4 ± 7.09	72.3 ± 12.9	20.1 ± 0.55	14.7 ± 1.65 ^b^	10.5 ± 2.32 ^b^
Psychological well-being, PWB ^a^, score	39.7 ± 0.11	37.7 ± 0.26 ^b^	37.8 ± 0.37 ^b^	38.1 ± 0.10	36.0 ± 0.40 ^b^	34.3 ± 0.64 ^b^
Ischemic heart disease % (n)	11.1 (287)	32.5 (172) ^b^	43.9 (105) ^b^	15.6 (547)	29.4 (87) ^b^	43.9 (47) ^b^
Stroke % (n)	2.7 (70)	8.4 (44) ^b^	10.5 (25) ^b^	2.9 (100)	5.1 (15) ^b^	10.5 (11) ^b^
Diabetes mellitus % (n)	6.0 (150)	14.7 (74) ^b^	19.4 (44) ^b^	6.5 (223)	19.9 (57) ^b^	29.8 (31) ^b^
Arterial hypertension % (n)	68.9 (1771)	80.1 (418) ^b^	82.6 (194) ^b^	56.0 (1957)	73.0 (214) ^b^	81.7 (85) ^b^
Body mass index, % (n)		*p* = 0.11			*p* < 0.001	
<25.0 kg/m^2^	22.8 (587)	26.1 (138)	21.3 (25)	23.8 (833)	14.2 (42) ^b^	16.8 (18)
25.0–29.9 kg/m^2^	45.3 (1166)	38.2 (202) ^b^	34.7 (83) ^b^	36.2 (1269)	30.5 (90) ^b^	25.2 (27)
>= 30.0 kg/m^2^	31.9 (821)	35.7 (189)	43.9 (105) ^b^	40.0 (1402)	55.3 (163) ^b^	57.9 (62) ^b^
Smoking habits % (n)		*p* < 0.001			*p* = 0.051	
Smokers	34.9 (895)	40.2 (211) ^b^	38.2 (91)	13.9 (487)	9.2 (27) ^b^	5.7 (6)
Former smokers	28.2 (724)	31.8 (167)	32.8 (78)	8.5 (298)	10.6 (31)	9.5 (10)
Never smokers	36.9 (948)	28.0 (147) ^b^	29.0 (69)	77.6 (2715)	80.1 (234)	84.8 (89)
Marital status % (n)		*p* < 0.001			*p* < 0.001	
Single	2.0 (51)	2.9 (15)	2.5 (6)	6.3 (220)	5.5 (16)	2.8 (3)
Married	84.8 (2176)	77.0 (405) ^b^	76.9 (183) ^b^	60.1 (2106)	50.2 (147) ^b^	50.9 (54)
Cohabiting	1.9 (49)	1.5 (8)	1.3 (3)	1.2 (42)	0.7 (2)	0.0 (0)
Divorced	8.0 (206)	10.5 (55)	9.7 (23)	17.1 (598)	18.1 (53)	16.0 (17)
Widowed	3.3 (84)	8.2 (43) ^b^	9.7 (23) ^b^	15.3 (537)	25.6 (75) ^b^	30.2 (32) ^b^
Education % (n)		*p* < 0.001			*p* < 0.001	
Primary	3.3 (84)	11.0 (58) ^b^	11.8 (28) ^b^	3.5 (123)	13.0 (38) ^b^	20.8 (22) ^b^
Vocational	7.6 (195)	15.0 (79) ^b^	13.4 (32) ^b^	6.1 (212)	11.3 (33) ^b^	10.4 (11)
Secondary	32.7 (839)	34.2 (180)	34.5 (82)	26.0 (910)	25.7 (75)	27.4 (29)
College	20.1 (206)	16.0 (84) ^b^	15.5 (37)	29.1 (1018)	23.3 (68) ^b^	20.8 (22)
University	36.3 (932)	23.8 (125) ^b^	24.8 (59) ^b^	35.4 (1240)	26.7 (78) ^b^	20.8 (22) ^b^
Depression scale score % (n)		*p* < 0.001			*p* = 0.101	
>= 4	14.3 (359)	21.3 (109) ^b^	23.7 (55) ^b^	27.9 (961)	32.5 (93)	36.5 (38)
<4	85.7 (2158)	78.7 (402) ^b^	76.3 (177) ^b^	72.1 (2485)	67.5 (193)	63.5 (66)
Cognitive function % (n)		*p* < 0.001			*p* < 0.001	
Normal	80.9 (2084)	73.0 (386) ^b^	69.5 (166) ^b^	89.2 (3129)	78.7 (233) ^b^	71.0 (76) ^b^
Lowered	19.1 (491)	27.0 (143) ^b^	30.5 (73) ^b^	10.8 (378)	21.3 (63) ^b^	29.0 (31) ^b^
PWB % (n)		*p* < 0.001			*p* < 0.001	
Higher	55.9 (1323)	41.7 (201) ^b^	41.6 (91) ^b^	58.2 (1894)	42.7 (114) ^b^	32.3 (31) ^b^
Lower	44.1 (1042)	58.3 (281) ^b^	58.4 (128) ^b^	41.8 (1358)	57.3 (153) ^b^	67.7 (65) ^b^

^a^ Age-adjusted means and standard errors. ^b^ *p* < 0.05 compared to the alive group separately for men and women, Bonferroni test. *p* values from χ^2^. ^c^ CVD deaths excluded those with previous CVD at the entry. SE: Standard Error. HDL: High-Density Lipoprotein. LDL: Low-Density Lipoprotein. PWB: Psychological Well-Being.

**Table 2:** Risk of all-cause and cardiovascular mortality* for a decrease in scores of cognitive functions ^a^ in men and women, Kaunas Health Alcohol Psychosocial Factors in Eastern Europe (HAPIEE) study, 2006–2017.

Cognitive Function/Cox		MEN				WOMEN		
Models	All-Cause	Deaths	CVD	Deaths	All-cause	Deaths	CVD	Deaths
		n = 667		n = 160		n = 384		n = 81
	Hazard Ratios (HR)	95% CI	HR	95% CI	HR	95% CI	HR	95% CI
Immediate verbal recall sum								
Model 1	1.19	1.10–1.29	1.26	1.08–1.47	1.26	1.12–1.41	1.46	1.15–1.84
Model 2	1.14	1.05–1.24	1.17	0.99–1.38	1.24	1.10–1.40	1.50	1.15–1.95
Model 3	1.16	1.06–1.27	1.18	0.99–1.40	1.25	1.10–1.41	1.70	1.29–2.24
Model 4	1.14	1.04–1.25	1.17	0.98–1.40	1.25	1.10–1.42	1.69	1.28–2.23
Delayed verbal recall								
Model 1	1.17	1.09–1.27	1.27	1.09–1.47	1.20	1.08–1.33	1.35	1.07–1.70
Model 2	1.12	1.04–1.23	1.17	0.99–1.38	1.19	1.06–1.33	1.46	1.14–1.88
Model 3	1.15	1.05–1.25	1.19	1.004–1.42	1.17	1.04–1.32	1.58	1.21–2.05
Model 4	1.13	1.04–1.23	1.19	1.003–1.42	1.17	1.04–1.32	1.57	1.21–2.04
Semantic verbal fluency								
Model 1	1.21	1.11–1.31	1.24	1.03–1.48	1.22	1.08–1.37	1.81	1.38–2.37
Model 2	1.16	1.07–1.27	1.16	0.96–1.39	1.19	1.06–1.34	1.81	1.37–2.40
Model 3	1.16	1.06–1.28	1.13	0.93–1.38	1.18	1.03–1.34	1.89	1.39–2.57
Model 4	1.15	1.05–1.27	1.14	0.94–1.38	1.18	1.04–1.34	1.00	0.76–1.31
Numerical ability								
Model 1	1.07	0.99–1.17	1.20	1.01–1.41	1.07	0.97–1.18	1.04	0.85–1.28
Model 2	1.03	0.94–1.13	1.09	0.90–1.31	1.08	0.97–1.20	1.11	0.89–1.38
Model 3	1.03	0.94–1.13	1.02	0.85–1.24	1.09	0.98–1.22	1.24	0.99–1.56
Model 4	1.03	0.94–1.13	1.03	0.85–1.24	1.09	0.97–1.21	1.24	0.99–1.56
Cognitive speed and attention								
Model 1	1.33	1.22–1.45	1.48	1.23–1.77	1.43	1.27–1.60	1.40	1.09–1.80
Model 2	1.29	1.17–1.41	1.39	1.14–1.70	1.37	1.21–1.54	1.43	1.10–1.87
Model 3	1.28	1.16–1.42	1.32	1.08–1.62	1.34	1.18–1.51	1.47	1.11–1.95
Model 4	1.24	1.12–1.37	1.32	1.08–1.62	1.33	1.17–1.51	1.00	0.77–1.31
Composite score of cognitive function								
Model 1	1.27	1.18–1.36	1.38	1.20–1.60	1.31	1.19–1.45	1.56	1.26–1.94
Model 2	1.20	1.11–1.30	1.24	1.06–1.46	1.30	1.17–1.44	1.68	1.31–2.14
Model 3	1.20	1.07–1.34	1.23	1.04–1.45	1.29	1.16–1.45	1.91	1.47–2.48
Model 4	1.19	1.10–1.30	1.32	1.08–1.62	1.30	1.16–1.45	1.91	1.48–2.48

* individuals with cardiovascular diseases (CVD) at the baseline survey removed from the analysis for CVD mortality. ^a^ per each 1 standard deviation decrease. HAPIEE: Health, Alcohol and Psychosocial factors In Eastern Europe. HR: Hazard Ratios. CI: Confidence Interval. Model 1 adjusted for age, education, and marital status. Model 2 adjusted for all the variables in Model 1 plus lifestyle (smoking, physical activity in leisure, alcohol consumption, and biologic factors (systolic or diastolic blood pressure, total cholesterol, high density lipoprotein (HDL) cholesterol, low density lipoprotein (LDL) cholesterol, triglycerides, fasting glucose, and body mass index (BMI)). Model 3 adjusted for all the variables in Model 2 plus depression symptoms and psychological well-being). Model 4 adjusted for all the variables in Model 3 plus existing illness (IHD, diabetes, and stroke—for all-cause mortality. Diabetes—for CVD mortality). For details, see Section 2.5. Statistical analysis. Bold typeface indicates significance.
